# Clinical Guidelines of the Egyptian Psychiatric Association for the Management of Treatment-Resistant Unipolar Depression in Egypt

**DOI:** 10.3389/fpsyt.2022.797150

**Published:** 2022-03-14

**Authors:** Momtaz Abdel-Wahab, Tarek Okasha, Mostafa Shaheen, Mohamed Nasr, Tarek Molokheya, Abd ElNasser Omar, Menan A. Rabie, Victor Samy, Hany Hamed, Mohamed Ali

**Affiliations:** ^1^Department of Psychiatry, Kasr El-Aini Medical School, Cairo University, Giza, Egypt; ^2^Okasha Institute of Psychiatry, Medical School, Ain Shams University, Cairo, Egypt; ^3^Department of Psychiatry, Kasr El-Aini Medical School, Cairo University, Cairo, Egypt; ^4^Department of Psychiatry, Alexandria Medical School, Alexandria University, Alexandria, Egypt; ^5^Department of Psychiatry, Ain Shams Medical School, Ain Shams University, Cairo, Egypt; ^6^Department of Psychiatry, Banha Medical School, Banha University, Benha, Egypt; ^7^Department of Psychiatry, Beni-Suef Medical School, Beni-Suef University, Beni-Suef, Egypt; ^8^Faculty of Pharmacy, German University of Cairo, Cairo, Egypt

**Keywords:** depression, treatment resistance, guidelines, clinical psychiatry, neuropharmacology

## Abstract

**Background:**

Major depressive disorder (MDD) is a public health burden that creates a strain not only on individuals, but also on the economy. Treatment-resistant depression in the course of major depressive disorder represents a clinically challenging condition that is defined as insufficient response to two or more antidepressant trails with antidepressants of the same or different classes that were administered at adequate daily doses for at least 4 weeks.

**Objective/Hypothesis:**

To develop a treatment guideline for Treatment Resistant Depression (TRD).

**Methodology:**

Experts in the field gathered and reviewed the available evidence about the subject. Then, a series of meetings were held to create recommendations that can be utilized by Egyptian psychiatrists.

**Results:**

The guidelines provide recommendations in various clinical settings. It evaluates different situations, such as patients at risk of resistance, those with resistance and recommends strategies to resolve the clinical case.

**Conclusion:**

The consensus guidelines will improve the outcomes of patients, as they provide recommendations across various domains that are of concern for the practicing psychiatrist.

## Introduction

MDD is defined as a disorder of having one or more major depressive episodes in a person's life with the absence of manic or hypomanic symptoms; to meet the criteria of MDD five out of nine symptoms, two of which must be low mood and anhedonia (loss of pleasure) persisting for a 2-week period. The nine symptoms include low mood, loss of interest or pleasure, disturbed sleep, change of eating pattern or weight, agitation or psychomotor slowness, unexplained fatigue, feeling worthless or guilty, inability to concentrate and thoughts of death or suicide (1).

Major depressive disorder (MDD) is a major public health concern. It is projected to be the first cause of disease burden worldwide by 2030 (2). According to the World Health Organization (WHO), depression affects over 260 million individuals worldwide (3). Results of a recent systematic review by Odejimi et al. reported that the prevalence of depression in Egypt ranges between 23.7 and 74.5% (4).

This mental disorder results from the interaction of several factors, including psychological, social and biological factors (5).

According to the Diagnostic and Statistical Manual of Mental Disorders, 5th Edition (DSM-5), a person is diagnosed with MDD when he or she consistently depressed mood or anhedonia, along with five of the following symptoms; difficulty in concentration, appetite changes, decreased energy, sleep disturbance, suicidal thoughts and tendencies, concentration difficulties, or psychomotor agitation or depression (6).

In medicine, the term “resistance” is used to describe the failure of standard treatment; although there is no distinct definition for TRD as clinical practice is ever evolving and because treatment “failure” is sometimes judged by physicians themselves when their patients do not respond as much as they expected them to. In short, TRD is described as depression that is unresponsive to antidepressant drug treatment at adequate dosing for an adequate amount of time (5).

Response to traditional treatment has been assessed by several studies (6). One major trial highlighted that the remission rate after step 1 of treatment was 36.8%, with the remission rate decreasing after each step of treatment, reaching only 13% at the fourth step (7, 8). In other words, the more advancement in treatment lines, the more likely is the patient to relapse. The same study concluded that the overall remission rate was 67%, highlighting that about 1 in every 3 patients fails treatment (9).

Several psycho-pharmacotherapeutic strategies have been suggested to overcome TRD, whereby augmentation treatment represents the currently recommended first-choice in case of insufficient response to the initial antidepressant treatment. In accordance with available international evidence, second-generation antipsychotics or lithium should be preferably employed (6).

Many patients are labeled as treatment resistant mistakenly when they are actually pseudo-resistant cases of depression; most common causes of this case are due to sub-therapeutic dosing or non-adherence to medication. According to APA guidelines it is important for physicians to assess any patient comorbidities, modifying doses of first line treatment if it failed after 4 weeks, considering patient history when adding second line treatment to ensure that the patient will positively respond to the medication and only initiating second line treatment after unsatisfactory response at 4 weeks (9).

“According to available international evidence, this publication will cover treatment options and recommendations from the Egyptian Psychiatric Association for the management of TRD in the course of MDD in Egypt.”

## Methods

Expert recommendations were determined after a thorough examination of available literature to identify and assess the recent global updates about the subject. Prior to the meeting, two senior members of the committee performed a comprehensive search on the PubMed database to identify the available literature relevant to the topic using keyword, such as “treatment-resistant depression”, “major depressive disorders” and their derivates. Then, those senior members prepared a questionnaire using the Delphi technique and invited committee members to assess the literature and respond to the questionnaire.

Afterwards, responses of clinicians to the questionnaire were captured, analyzed and ranked. Then, another round of questions was dispensed in a second meeting based on the outcomes of the first committee gathering and questionnaire responses. The second round was when a consensus was reached by all committee members, therefore there was no need for further meetings (10).

## Results

### Panel Description

An expert panel of 8 professors of psychiatry from different universities and the ministry of health representing the Egyptian Psychiatric Association drafted the following guidelines. Information about panel members can be found in the Appendix.

### Definition of Resistant Depression

According to a meta-analysis by Gaynes et al. 2019, there is no clear-cut definition for TRD, however, most experts unanimously agree that lack of response to initial treatment is deemed as TRD. The APA (American Psychological Association) and NICE (National Institute for Health and Care Excellence) guidelines also state that the next step of handling TRD also differs between experts which makes it more challenging to develop universal treatment guidelines. The classes of medications should be among the following: (11).

- Tricyclic antidepressants (TCAs) (equivalent to 300 mg imipramine) (12)
- Selective serotonin reuptake inhibitors (SSRIs) (equivalent to 50 mg fluoxetine) (13)
- Serotonin norepinephrine reuptake inhibitors (SNRIs) (equivalent to 225 mg venlafaxine) (14)

Antidepressant treatment has been the mainstay treatment of depression for years; the first class of such drugs were tricyclic antidepressants followed by more novel agents namely SSRIs and SNRIs. Both classes of TCA and SSRIs are equally affective in ameliorating depressive symptoms and decreasing depression scores as proved by a meta-analysis to compare the two classes. However, SSRIs are preferred by patients as well as physicians as they do not produce bothersome side effects as TCAs (15).

SNRIs are a group of antidepressants with a dual mechanism of action of both serotonin and adrenergic reuptake inhibition; research has suggested that SNRIs are superior to SSRIs in more severe depression; which insinuates their possible effectiveness in TRD. Moreover, SNRIs have established efficacy in treating depression with somatic manifestations as pain and other physical symptoms (16). The Danish guidelines; which incorporates the APA and NICE guidelines states that most patients first diagnosed with MDD are treated with SSRIs then switched to SNRIs. The ten most prescribed treatments for TRD mainly constitute of both SSRI's and SNRI's as first, second- and third-line treatments (17). Other antidepressants as trazodone, vilazodone and tranylcypromine are also available options however, are not easily accessible in the Egyptian market.

The advisory committee recommended of TRD be as follows:

Treatment-resistant depression shall be defined as the failure of 2 different classes of antidepressant medications, given that the medications have been used for a period of 6–8 weeks at the desired dose.

N.B. (Nota bene) to the above definition and in order to account for the definition of TRD in Egypt, the committee added 6–12 monitored sessions of BST (Brain Synchronization Therapy)/ECT (Electroconvulsive Therapy).

The panel recognized the importance of identifying the term “pseudo-resistance”, which was defined by the panel as a patient not responding to medication because of a problem in the diagnosis or the type of depression or the presence of depression secondary to another psychiatric, personality or medical disorder.

In addition to pseudo-resistance, the clinical experts highlighted additional predictors for TRD, as follows:

- Other forms of depression such as bipolar depression.
- Other comorbid conditions, including concurrent anxiety, drug abuse, chronic organic medical conditions, and personality-related disorders (e.g., mood swings in borderline personality disorder).

### Assessment of Treatment-Resistant Depression

Clinical panel experts recommended using the following set of clinical tools and tests to identify and diagnose TRD.

- Depression severity scales such as Montgomery-Asberg Depression Rating Scale (MADRS) and psychiatrist-rated scales. Montgomery-Asberg Depression Rating Scale has long been used by physicians for assessing depression, the advantage of MADRS is that avoids the drawbacks of the HAM-D score and is more robust. It is used mainly to detect any patients' response changes to antidepressant therapy with high sensitivity and is positively correlated to change in degree of depression (18).
- Hypomania check list: The hypomania checklist has long been used in many countries and in different languages to differentiate bipolar depression from MDD. This is important as manic/psychotic symptoms in depression should not be confused with TRD (19).
- Suicide scale [e.g., Beck's Scale for Suicide Ideation or Columbia Suicide Severity Rating Scale (C-SSRS)]. Suicide ideation scales are mainly used to monitor patient's health and to predict the risk of actual suicide for timely intervention (20). The BSS is one of the most reliable tools to predict a patient's risk and plan for suicide. The C-SSRS is also a sensitive scale to use especially that it is sensitive to change of suicidal ideation over treatment time and with the use of medication (21).
- Complete blood count (CBC), liver and kidney function tests, lipid and glycemic profiles: Complete blood count (CBC), liver and kidney function test and lipid and glycemic profiles; all these tests can be used and are done to eliminate any disease that could precipitate symptoms of depression (22).
- Thyroid function test: Regarding thyroid profiling; it is established through a wide volume of research that hypothyroidism presents with some depressive symptoms, in a study conducted by Bathla et al. 56% of males and 64% of females with hypothyroidism presented with some symptoms of depression and anxiety (23).
- Electrocardiogram: An electrocardiogram could be beneficial since stress (a component of MDD) is linked to heart disease. Not only that; depression onset is often seen in up to 40% of patients after a major cardiac event. Therefore, an ECG could be of use to determine heart health of patients and to stratify patients who could be at risk of developing MDD or TRD (24).
- Brain imaging techniques like magnetic resonance imaging (MRI): Recently, brain imaging using MRIs or CT scans have been utilized in the diagnosis of MDD especially in the elderly. This is because some cases of depression indicate an underlying mental disease as Parkinson's, Alzheimer's and Pick's disease; moreover, geriatric depression has been associated with leukoencephalopathy that can be detected using brain imaging techniques (25).

The panel highlighted the importance of periodic performance of most of the above tests upon prescribing ADs. In addition to the aforementioned tests, the panel also recommends performing the following tests based on clinician's discretion and the patient profile, as follows:

- Assessing vitamin D plasma levels
- Assessing sex hormones plasma levels
- Toxicological analysis of blood and urine samples

### Principles of Management

#### Hospitalization: Indications

Upon careful assessment of the condition, the expert panel agreed that psychiatric hospitalization is warranted in severe cases that fall under the umbrella of one of the following:

- Catatonic cases
- Patients with high suicidal tendency
- Severe psychotic symptoms
- Advanced cases of MDD
- The presence of severe uncontrolled comorbid medical conditions
- Insufficient familial support to the patient

### Therapeutic Options for Treatment-Resistant Depression and Comorbid Conditions

#### Pharmacotherapeutic Options of Comorbid Conditions

For patients suffering from comorbid conditions, the expert panel recommends the following:

Patients suffering from anxiety and its features are recommended to receive benzodiazepines. Buspirone and pregabalin are also available options. Anxiety and depression often go hand in hand; therefore, a multi-modal treatment approach for handling both illnesses are often recommended. The evidence in the treatment of anxiety disorders greatly points to SSRIs, SNRIs and benzodiazepines. Benzodiazepines have long been used as anxiolytics as they demonstrate a relatively high safety and tolerability profile, they also have a rapid onset of action and manage acute anxiety symptoms and somatic complaints according to the NICE guidelines. The common issue physicians and patients face with benzodiazepines is dependence and abuse; thus, benzodiazepines should be preferentially used for a short amount of time (26).

The NICE guidelines usually recommend pregabalin in patients with anxiety if they do not tolerate SSRIs and SNRIs; pregabalin is an anti-convulsant and is also used for neuropathic pain; the advantage of pregabalin over benzodiazepines is that it does not cause dependance; however, sudden discontinuation may cause confusion. Buspirone, a non-benzodiazepine alternative is also prescribed for anxiety symptoms; the evidence regarding its efficacy in comparison to benzodiazepines is conflicting; however, it does not cause sedation, withdrawal symptoms or addiction as benzodiazepines (27).

Patients with depression or TRD frequently suffer from sleep disturbances; therefore, a sleep-aid can be used to overcome this symptom. Benzodiazepines; although excellent sleep-aids; often cause residual daytime sleepiness or “hangover”, high doses of benzodiazepines also cause cognitive impairment and respiratory depression therefore, pose a risk; in contrast to hypnotic agent zolpidem that lacks effect of residual day-time sedation or psycho-motor impairment. Zopiclone, another hypnotic agent; is also free of any day-time sleepiness side effect but slightly impairs psychomotor function especially at high doses. Both agents are reported to be tolerated, efficacious, safe and have low rates of dependence or abuse (28).

Other non-benzodiazepines that also act as sedatives are trazodone (an antidepressant from a class of serotonin modulators) and low dose quetiapine (an atypical anti-psychotic). Quetiapine has a wide array of indications; it has the advantage of ameliorating anxiety symptoms so can be used in TRD comorbid with anxiety, chronic pain and PTSD. In depression; 27 patients in a MDD episode were administered low dose quetiapine along with venlafaxine or escitalopram, by the end of the 4 weeks test time; sleep parameters had definitely improved in all patients (29). A head-to-head comparative study between trazodone and low dose quetiapine in hospitalized psychiatric patients at St. Helena's Hospital suffering from insomnia revealed that trazodone is a superior agent in patients with depressive symptoms and offers higher improvement in sleep parameters than quetiapine (30).

Patients at high risk of self-inflicted injury are recommended to receive lithium, benzodiazepine, or second-generation antipsychotic medication. This is because second benzodiazepines generation anti-psychotics as clonazepam have a mood stabilizing effect and reduce impulsivity and mood swings (31).

### Treatment Duration

The clinical committee members recommended that patients should be maintained on their ongoing antidepressant medication for a period of 9–12 months following the achievement of clinical remission.

Patients suffering from the following conditions are recommended to receive a longer course of treatment:

- Long period to reach remission
- History of 2 prior depression episodes
- History of early relapse after treatment discontinuation
- Presence of suicidal tendency, symptoms of psychosis, family history of suicide or mood disorders or comorbid psychiatric condition.
- Resistance to an antidepressant medication when given at proper dose and adequate duration.

### Therapeutic Options of Patients Suffering From TRD

**Figure 1:**
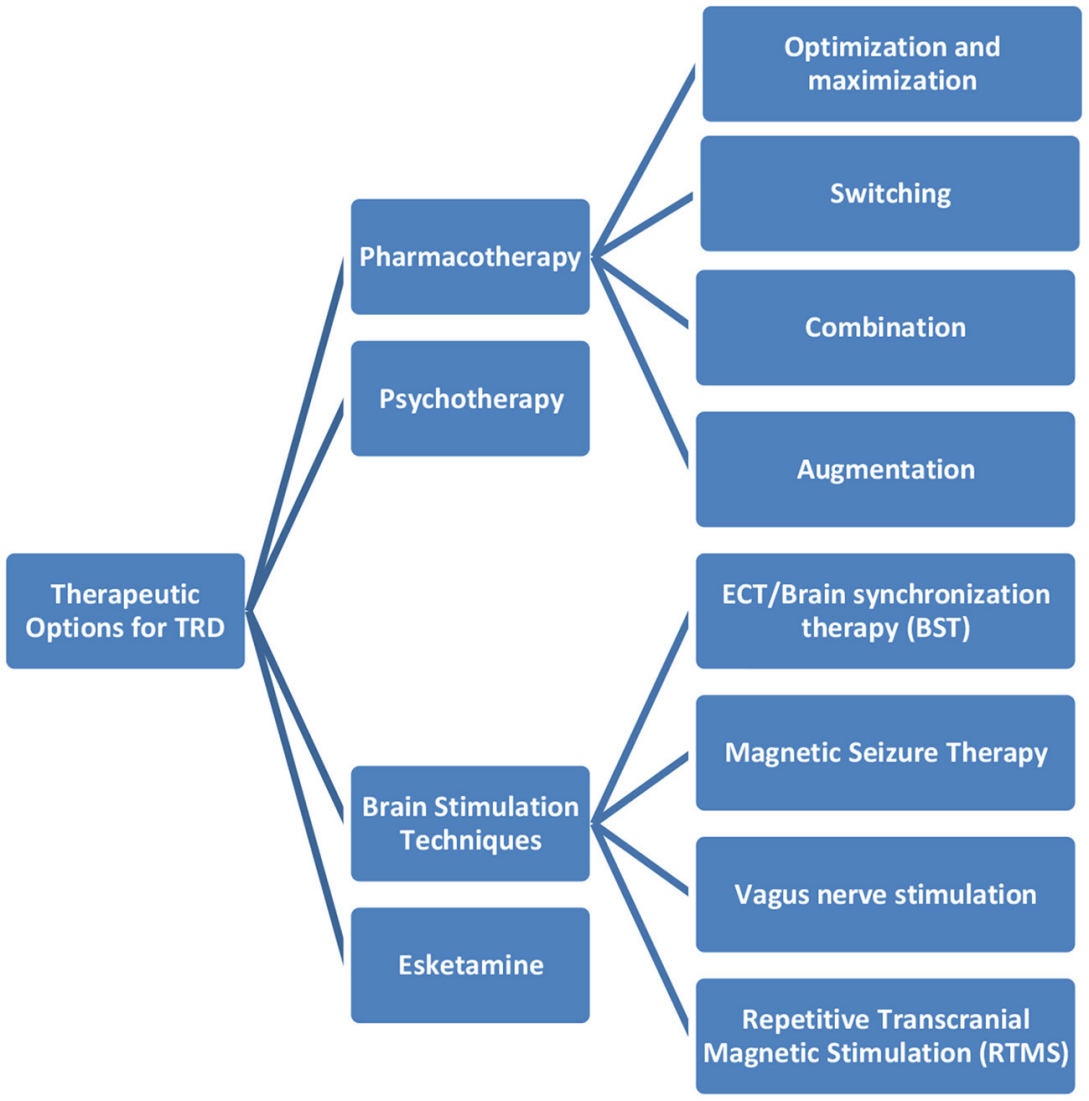
The available therapeutic options for TRD.

The panel experts highlighted the available treatment options for patients suffering from TRD. The available therapeutic options are captured in Figure 1.

### Pharmacological Strategies in Treatment-Resistant Depression

#### Switching Strategies

Switching involves shifting to another antidepressant medication, either within the same class or from a different class. Switching to a medication within the same class is undertaken to obtain a different pharmacological property, while switching to another class usually yields a different neurochemical effect. This strategy is tailored to suit individual patient needs and preferences (5).

According to evidence gathered about switching treatment in TRD; switching has certain pros over augmentation therapy; first it carries lower risk of drug-drug interactions, has higher patient adherence, moreover, it is preferred for patients who suffered severe side effects from first line medication and displayed partial or no response.

Only two major trials have been conducted to observe switching of medications in TRD, the first was about patients who had previously failed two antidepressants (mostly SSRIs) and were switched to either SSRI paroxetine or SNRI venlafaxine; response rates were 33 and 52%, respectively while remission rates were 20 and 42%. Other studies involved switching from SSRI to extended-release venlafaxine and switching from an SSRI to mirtazapine or sertraline, both studies showed no significant difference in depressive symptoms.

The panel experts recommend switching to be carried out in the following situations:

- Lack of response or poor tolerance to initial treatment
- Prior response to the introduced medication

**Figure 2:**
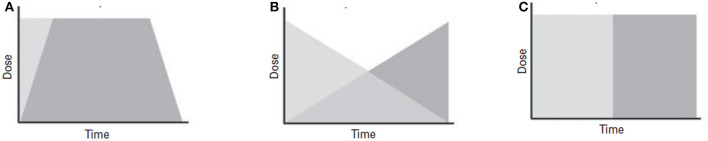
Schematic diagrams of switching strategies. **(A)** Concurrent switch is best suited for patients demonstrating partial response, where simultaneous change in the dose of both medications is implemented. **(B)** Overlapping switch is suitable for patients who demonstrate partial response, where the dose of the original medication is maintained until the second medication reaches its optimal dose. **(C)** Sequential switch is considered to be the safest switching technique, as it is the least likely to cause any interaction. In this strategy, one medication is substituted with another. This technique is used in patients who do not respond to initial treatment.

The clinical experts highlighted 3 different types of switching strategies, with concurrent switching being the most recommended, except for medications belonging to monoamine oxidase inhibitor (MAOIs) class. These strategies are captured in Figure 2 (32).

**Figure 3:**
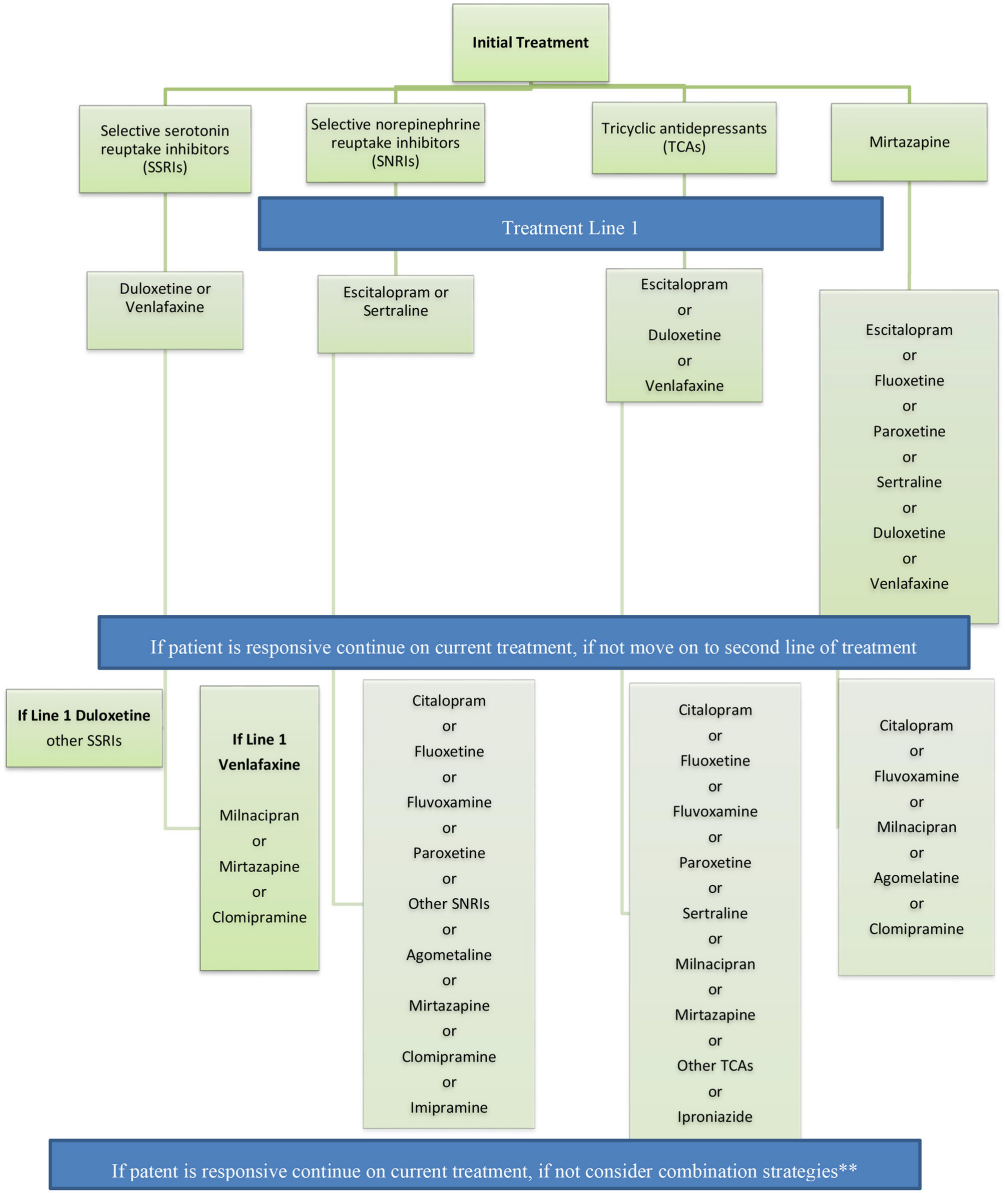
Recommendations for switching antidepressant medications. If patient is responsive continue on current treatment, if not consider combination strategies** Adapted from the French guidelines. ^**^Combination strategy = The panel recommends the use of combination strategy in patients with partial response after adequate treatment with a medication for a period of 2–4 weeks.

Recommendations for switching antidepressant medications are captured in Figure 3.

### Combination Strategies

Combination strategies usually involve using 2 different antidepressant medications belonging to different classes and having different pharmacodynamic profiles (33).

The panel recommends the use of combination strategy in patients with partial response after adequate treatment with a medication for a period of 2–4 weeks (4–6 weeks if with TCAs).

The recommended first-line combination strategy involves mirtazapine plus one of the following:

- Selective serotonin reuptake inhibitor.
- Serotonin–norepinephrine reuptake inhibitor.
- Tricyclic antidepressant.

### Augmentation Strategies

Augmentation strategies refers to the addition of non-standard antidepressant medications, like lithium and quetiapine, to enhance the outcome of classical antidepressants (34). The panel summarized the medications and lines of choice in Table 1.

**Table 1:** Therapeutic lines in augmentation strategy in non-psychotic patients.

**Potentiation treatment**
**First choice**
• Lithium with serum level must be at least 0.8 mmol/L
• Second generation anti-psychotic with antidepressant action (Quetiapine, Asenapine, Iloperidone, Brexpiprazole, Laurasidone, Cariprazine)
**Second choice**
• Aripiprazole
•Tri-iodothyronine
• Lamotrigine

The panel recommends this strategy in patients who demonstrate partial response after 2–4 weeks of treatment (4–6 weeks with TCA as they have a delayed effect than newer agents).

The panel recommends adding lithium or quetiapine to improve efficacy of the antidepressant medication.

The panel recommends a second choice of thyroid hormone supplementation in addition to serotonin–norepinephrine reuptake inhibitors or tricyclic antidepressants, and with selective serotonin reuptake inhibitors or mirtazapine at a later stage.

The recommended dose of thyroid hormone supplementation is between 25 and 60 ug/day of liothyronine (L-T3) and is required to achieve TSH levels ranging between 0.1 and 1 ug/L. Prior to initiating treatment, the panel recommends performing the following assessments:

- Physical examination
- Electrocardiogram (ECG)
- Thyroid-stimulating hormone (TSH) levels.

### Treatment Sequence for Depression Dimensions

Major depressive disorder is a multidimensional disorder that originates from multiple etiologies. The symptom dimension may act as predictor of antidepressant treatment response (35).

The panel recommendations for the first and second lines of treatment of several depression dimensions are illustrated in Table 2.

**Table 2:** Treatment lines recommendations for clinical dimensions in MDD.

**Dimension**	**First choice**
With marked anhedonia	NDRI or SNRI
With marked psychomotor retardation	SNRI or NDRI
With marked sleep disturbances	SSRI or SNRI or Mirtazapine or agomelatine
With atypical features (hyperphagia, hypersomnia)	SSRI or SNRI
With psychotic features	SNRI in monotherapy or SSRI in combination with an atypical 2nd generation antipsychotic with an antidepressant action
With anxious features	SSRI or SNRI or Mirtazapine or Lithium
With high suicidal risk	SSRI or SNRI or Mirtazapine or Lithium or 2nd generation antipsychotic with an antidepressant action
Positive family history of bipolar disorder or suicide	Mood stabilizer (Lithium)

### Brain Stimulation Techniques

The panel selected ECT/BST, and repetitive transcranial magnetic stimulation (rTMS) as the preferred brain stimulation techniques that are either used alone or in combination with antidepressants. The panel agreed that ECT/BST represent effective approaches to prevent relapse, either alone or in combination. Recommendations of brain stimulation techniques are as follows:

BST is recommended for resistant cases after failure of 3 adequately used antidepressants.

### Novel Therapeutic Agents

#### Esketamine

Esketamine, the S enantiomer of ketamine, is a N-methyl-D-aspartate (NMDA) receptor antagonist that has higher affinity to the receptor compared to the R enantiomer and the racemic mixture of ketamine (36). In the form of a nasal spray, this molecule has recently gained approval in the United States for the indication of TRD as well as by the EMA in Europe for the indication of TRD, bringing hope to patients who suffer from the condition (37).

Esketamine, in combination with SSRIs or SNRIs, is indicated for the treatment of TRD in patients who did not respond to least 2 different classes of antidepressant medications (38).

### Strategies to Prevent Relapse

A relapse is the return of depressive symptoms to patients. Relapse could be early or delayed. In the former, symptoms return is expected to be within the first 3–12 months, while the latter refers to the emergence of new depressive episodes following remission or initial short-term improvement in symptoms (39).

It is of importance to have measures to prevent relapse from the first relapse episode. With regard to preventive strategies, the following has been highlighted:

The panel recommends the use of ECT and lithium as effective first-line options to prevent relapse.

The panel recommends the use of lamotrigine or quetiapine as second-line options to prevent relapse.

The panel recommends the following strategies when patients achieve full remission:

- Continuous assessment of patients' adherence to treatment.
- Continuous assessment of social functioning of patients.
- Continuous assessment of quality of life (QoL) of patients.

The panel recommends the following complementary approaches for relapse and recurrence prevention:

- Undertaking regular physical exercises and activities.
- Eating healthy food
- Control physical illness (e.g., hypertension, diabetes, etc.…)

## Discussion

Treatment resistant depression is regarded as patients failing 2 subsequent antidepressant treatments (38); in Egypt TRD's definition is slightly altered to the latter by the addition of failure of 6–12 sessions of ECT or BST (Brain Stimulation Techniques). Approximately 60–70% of depressed patients do not respond to first line treatment and more than a third become treatment resistant. However, TRD should not be confused with “pseudo-resistance”. This means that patient-related factors that might contribute treatment failure should be taken into account before deeming their depression as “resistant”. These factors are numerous, however inadequate dosing, compliance, follow-up and primary mis-diagnosis are prime examples (38). Another review also added other patient traits; the presence of comorbid psychiatric illnesses as OCD (Obsessive Compulsive Disorder), bipolarity, anxiety and eating disorders as well as the presence of psycho-somatic disorders as Fibromyalgia and IBS (Irritable Bowel Syndrome), all of which must be assessed carefully (with several available diagnostic tools) as they may lead to high rates of depression recurrence and severity; higher severity of depressive illness and the higher rates of relapses in between remission periods is definitely a precipitating factor for TRD (39).

The panel urges that patients are monitored for both psychiatric and somatic diseases; this is reinforced by Kornstein's data who reported that patients with hypothyroidism and depression had higher remission rates when properly treated for under-active thyroid, this agrees with the panel's recommendation in augmenting therapy with T3 hormone supplementation. Patient history and concurrent medications must be known as some drugs such as gluco-corticosteroids and anti-inflammatories are associated with causing depressive symptoms (39). Ergo, the panel suggests using a list of tools and tests as MRIs, blood work, organ function tests etc. to monitor for certain patient conditions that may cause depression treatment failure or increase severity of the illness.

The panel agrees with the international guidelines for hospitalization indications in patients with TRD, this is because hospitalization prevents further complications that may be caused if TRD and/or its comorbidities are not under continuous medical supervision (8). A study conducted in Finland on a population of hospitalized patients as a result of depression followed-up patients up to 24 years after discharge to evaluate their outcomes. Of about 15,000 patients followed-up; only 2,567 died by suicide with an overall cumulative risk of suicide of 6.13%; which is considerably lower than depressed patients who remain unhospitalized (40).

Regarding comorbid mental illnesses with depression the panel recommends using several drugs in addition to antidepressants; the anxiolytics used is in line with the Canadian clinical practice guidelines; generally, benzodiazepines are fit for all anxiety disorders; Buspirone is indicated for a wide range of anxiety diseases as panic disorder, social anxiety, OCD (Obsessive Compulsive Disorder), GAD (Generalized Anxiety Disorder) and PTSD (Post-traumatic Stress Disorder), Pregabalin however, is used as second line treatment if first line drugs are not tolerated. For sleep disorders and self-harm comorbid with TRD, the panel's recommendations are based on clinical experience; which of course at times conflicts with published research. A Cochrane meta-analysis had similar documented patients who were prescribed drugs similar to the panel's recommendations as atypical anti-psychotics and benzodiazepines for both self-harm and sleep disorders (41).

Physicians however, must be aware of which drug to add to the patient's treatment plan and patient monitoring and continuous follow-up is crucial to their wellbeing. In Table 3, the panel has several recommendations of which medications to prescribe if TRD were associated with any other clinical issue such as sleep disturbances, suicide ideation, anxious features etc. Mirtazapine is an excellent antidepressant with a wide array of indications (and off-label uses) and has demonstrated superiority to tricyclic antidepressants due to its lack of any anti-cholinergic, adrenergic and serotonin-mediated side effects; clinical evidence has proved that it has transferred the treatment of depression. In comparison to tricyclic antidepressants as amitriptyline, clomipramine, doxepin and serotonin modulator trazodone, the clinical effect of mirtazapine was similar and at times superior, not only that, but it is also strongly advocated in cases of depression with poor sleep scores in which mirtazapine improved drastically in comparison to placebo. According to these findings, mirtazapine's adverse effects as dry mouth, increased appetite and weight gain should be weighed together with its high safety and effective treatment profile, when exploring other drugs to prescribe/augment/combine with the current treatment profile (42).

**Table 3:** The main findings of this study.

**Area of interest**		**Recommendation**
Hospitalization: indications		The panel members recommended the consideration of psychiatric hospitalization for the following cases. - Catatonia - Patients with high suicidal tendency - Severe psychotic symptoms - Advanced cases of MDD - The presence of severe uncontrolled comorbid medical conditions - Insufficient familial support to the patient
Therapeutic options for treatment -resistant depression and comorbid conditions pharmacotherapeutic options of comorbid conditions		For patients suffering from comorbid conditions, the expert panel recommends the following: Patients suffering from anxiety and its features are recommended to receive benzodiazepines. Buspirone and pregabalin are also available options. Patients suffering from sleep disorders are recommended to receive adjunctive hypnotic medications, such as zolpidem or zopiclone. Patients at high risk of self - inflicted injury are recommended to receive lithium, benzodiazepine, or second -generation antipsychotic medication.
Treatment duration		The clinical committee members recommended that patients are maintained on their ongoing antidepressant medication for a period of 9–12 months following the achievement of clinical remission. Patients suffering from the following conditions are recommended to receive a longer course of treatment: - Long period to reach remission - History of 2 prior depression episodes - History of early relapse after treatment discontinuation - Presence of suicidal tendency, symptoms of psychosis, family history of suicide or mood disorders or comorbid psychiatric condition. - Resistance to an antidepressant medication when given at proper dose and adequate duration.
Therapeutic options of patients suffering from TRD		The panel experts highlighted the available treatment options for patients suffering from TRD. The available therapeutic options are captured in Figure 1.
**Pharmacological strategies in treatment-resistant depression**		
Switching strategies		The panel experts recommend switching to be carried out in the following situations: - Lack of response or poor tolerance to initial treatment - Prior response to the introduced medication The clinical experts highlighted 3 different types of switching strategies, with concurrent switching being the most recommended, except for medications belonging to monoamine oxidase inhibitor (MAOIs) class. These strategies are captured in Figure 2 (32).
		Recommendations for switching antidepressant medications are captured in Figure 3.
^**^Combination strategies		The panel recommends the use of combination strategy in patients with partial response after adequate treatment with a medication for a period of 2–4 weeks (4–6 weeks with TCAs). The recommended first-line combination strategy involves Mirtazapine plus one of the following: - Selective serotonin reuptake inhibitor. - Serotonin–norepinephrine reuptake inhibitor. - Tricyclic antidepressant.
Augmentation strategies		The panel recommends this strategy in patients who demonstrate partial response after 2–4 weeks of treatment (4–6 weeks if on TCAs). The panel recommends adding lithium or quetiapine to improve efficacy of the antidepressant medication. The panel recommends a second choice of thyroid hormone supplementation in addition to serotonin–norepinephrine reuptake inhibitors or tricyclic antidepressants, and with selective serotonin reuptake inhibitors or mirtazapine at a later stage. The recommended dose of thyroid hormone supplementation is between 25–60 ug/day of liothyronine (L-T3) and is required to achieve TSH levels ranging between 0.1 and 1 ug/L. Prior to initiating treatment, the panel recommends performing the following assessments: - Physical examination - Electrocardiogram (ECG) - Thyroid-stimulating hormone (TSH) levels (Table 1).
Treatment sequence for depression dimensions		The panel recommendations for the first and second lines of treatment of several depression dimensions are illustrated in Table 2.
Brain stimulation techniques (BST)		The panel selected ECT/BST, and repetitive transcranial magnetic stimulation (rTMS) as the preferred brain stimulation techniques that are either used alone or in combination with antidepressants. The panel agreed that ECT/BST represent effective approaches to prevent relapse, either alone or in combination. Recommendations of Brain Stimulation Techniques are as follows: BST is recommended for resistant cases after failure of 3 adequately used antidepressants.
Novel therapeutic agents		Esketamine, in combination with SSRIs or SNRIs, is indicated for the treatment of TRD in patients who did not respond to least 2 different classes of antidepressant medications (38).
Strategies to prevent relapse		It is of importance to have measures to prevent relapse from the first relapse episode. With regard to preventive strategies, the following has been highlighted:
		The panel recommends the use of ECT and lithium as effective first-line options to prevent relapse. The panel recommends the use of lamotrigine or quetiapine as second-line options to prevent relapse. The panel recommends the following strategies when patients achieve full remission: - Continuous assessment of patients' adherence to treatment. - Continuous assessment of social functioning of patients. - Continuous assessment of quality of life (QoL) of patients. The panel recommends the following complementary approaches for relapse recurrence prevention: - Undertaking regular physical exercises and activities. - Eating healthy food - Control physical illness (e.g., hypertension, diabetes, etc.…)

Another interesting agent that can be used and is recommended by Egyptian psychiatrists is agomelatine, one of the first melatonergic agents and a 5-hydroxytryptamine receptor (5-HT_2C_) antagonist both of which act harmoniously to adjust disrupted circadian rhythms (sleep cycles) typically found in depressive illness. As mentioned above, depressed patients must be closely monitored to avoid any consequences unintentionally caused by treatment; agomelatine should not be used in patients with a compromised liver and in healthy patients, liver function tests must be routinely done at the beginning of treatment and subsequently at 6 weeks, 12 weeks and 6 months as recommended by the EMA (European Medicines Agency) (43).

Moving forward, a pharmacological treatment algorithm was devised as mentioned above in Figure 3; there are several strategies that physicians can adhere to when approaching TRD, these include switching, combination or augmentation strategies. A study in 2001 evaluated patients wth low response to Fluoxetine 20 mg/day who were switched to mianserin 60 mg/day. The results were intermediate but were still sound with depression scores lower by 1.8 in the mianserin group than those who continued on Fluoxetine. The same study also had a third treatment arm with patients on fluoxetine therapy combined with mianserin (=combination strategy) in which the depression score plummeted by 4.6 in the combined treatment group; this is a commonly observed phenomenon in combination strategies as both drugs synergistically act to create a larger overall effect in managing symptoms especially of a multi-modal disease as depression (44).

Furthermore, augmentation strategy is adding a different class of medication to a current antidepressant; this was done in a study where randomized depressed patients who remitted from ECT.They received placebo, lithium or lithium with nortriptyline; 84% relapsed on placebo, 60% on lithium monotherapy and 39.1% on the combination therapy which consolidates the fact that combination therapy with non-antidepressant agent proves useful in treatment of depression as well as preventing relapses (45). There is also evidence that suggests that Lithium decreases the risk of suicide in depressed patients (46).

There are other methods of treatment in TRD to resort to such as ECT (a brain stimulation technique) or the use of esketamine (a relatively novel therapeutic agent). ECT therapy has long been recommended by the British guidelines of 2000 for severe cases of depression especially those who have failed two or more drugs (TRD), rTMS is recommended second to ECT but must be done by a team of specialists. Ghasemi made a more interesting discovery when comparing patients who received three sessions of ECT vs. those who received low dose esketamine over 3 days; results supported that both treatments although comparable, esketamine had a more rapid and more pronounced resolution of symptoms (47). The use of esketamine is not yet approved in Egypt; although it has long been approved by the FDA in the United States and the EMA in Europe for its relative safety and efficacy; moreover, adverse effects of dissociation, vertigo and dizziness from esketamine usually resolved on the same day, shortly after administration; (48) future-wise; the use of esketamine (only as nasal spray) must be warranted in Egypt due to its pronounced effect with patients feeling better within hours of administration and lower relapse rates as maintenance treatment all of which is vital for a TRD patient.

Following achieving remission for depression; a major challenge is to prevent relapse of depressive episodes; the use of lithium is widely agreed upon in patients who were suicidal, ECT is proposed for patients with frequent relapses. Physiological wellbeing is also an aspect to be considered; any emerging or residual mental or physical illness should be tended to in order to prevent relapse (46).

Like all studies, this study carries its own strengths and limitations. Surely the first strength of this study is that this is the first consensus of guidelines between Egyptian psychiatrists for the treatment of TRD; the panel's recommendations came from practicing them on TRD patients in an expert clinical setting. The limitation of this study is that the panel constituted of only eight doctors who only represent a small group of psychiatrists from a much larger number in Egypt; additionally, there is no scientifically exact or official definition of TRD or for its treatment of TRD in Egypt or world-wide (internationally) to base these recommendations on; they were merely observations collected by the panel experts.

## Conclusion

The integration of clinical practice with the latest updates of clinical studies yields the best outcomes for patients. TRD puts a significant burden on the patient, therefore, it is always best to manage the condition with careful review and step-wise approach. The development of recent pharmaceutical options for patients with TRD ushers a new area to tackle this condition.

## Data Availability Statement

The original contributions presented in the study are included in the article/Supplementary Material, further inquiries can be directed to the corresponding author/s.

## Author Contributions

All authors listed have made a substantial, direct, and intellectual contribution to the work, participated in its writing and approved it for publication.

## Conflict of Interest

The authors declare that the research was conducted in the absence of any commercial or financial relationships that could be construed as a potential conflict of interest.

## Publisher's Note

All claims expressed in this article are solely those of the authors and do not necessarily represent those of their affiliated organizations, or those of the publisher, the editors and the reviewers. Any product that may be evaluated in this article, or claim that may be made by its manufacturer, is not guaranteed or endorsed by the publisher.
