# Supplementary material for: Clinical Guidelines of the Egyptian Psychiatric Association for the Management of Treatment-Resistant Unipolar Depression in Egypt
Source: Front Psychiatry. 2022 Mar 14;13:797150. doi: 10.3389/fpsyt.2022.797150 (PMC8964405; doi:10.3389/fpsyt.2022.797150)
Supplement: Supplementary file 1 [file Presentation_1.PPTX]

## Slide 1
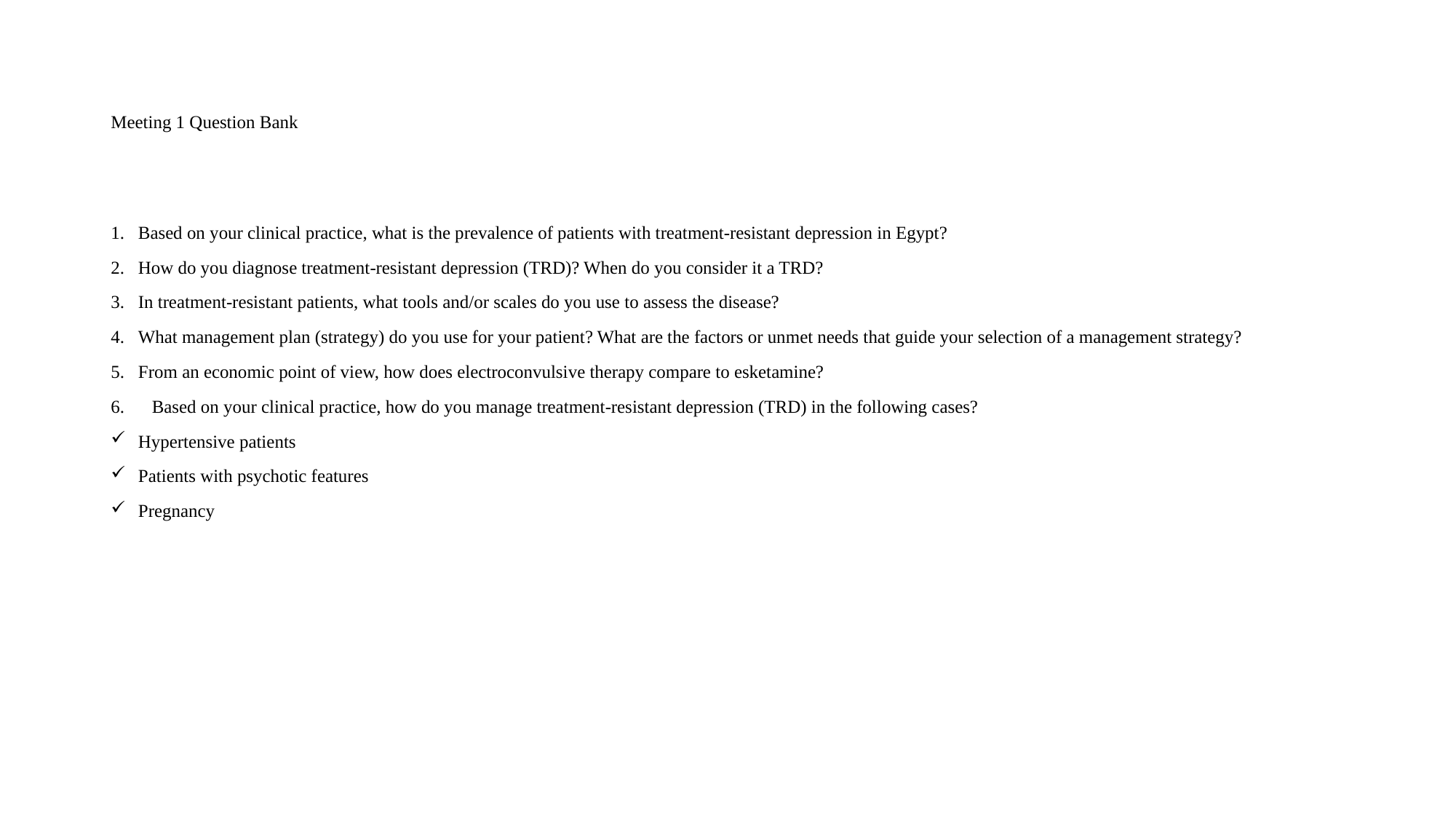

# Meeting 1 Question Bank
Based on your clinical practice, what is the prevalence of patients with treatment-resistant depression in Egypt?
How do you diagnose treatment-resistant depression (TRD)? When do you consider it a TRD?
In treatment-resistant patients, what tools and/or scales do you use to assess the disease?
What management plan (strategy) do you use for your patient? What are the factors or unmet needs that guide your selection of a management strategy?
From an economic point of view, how does electroconvulsive therapy compare to esketamine?
Based on your clinical practice, how do you manage treatment-resistant depression (TRD) in the following cases?
Hypertensive patients
Patients with psychotic features
Pregnancy

## Slide 2
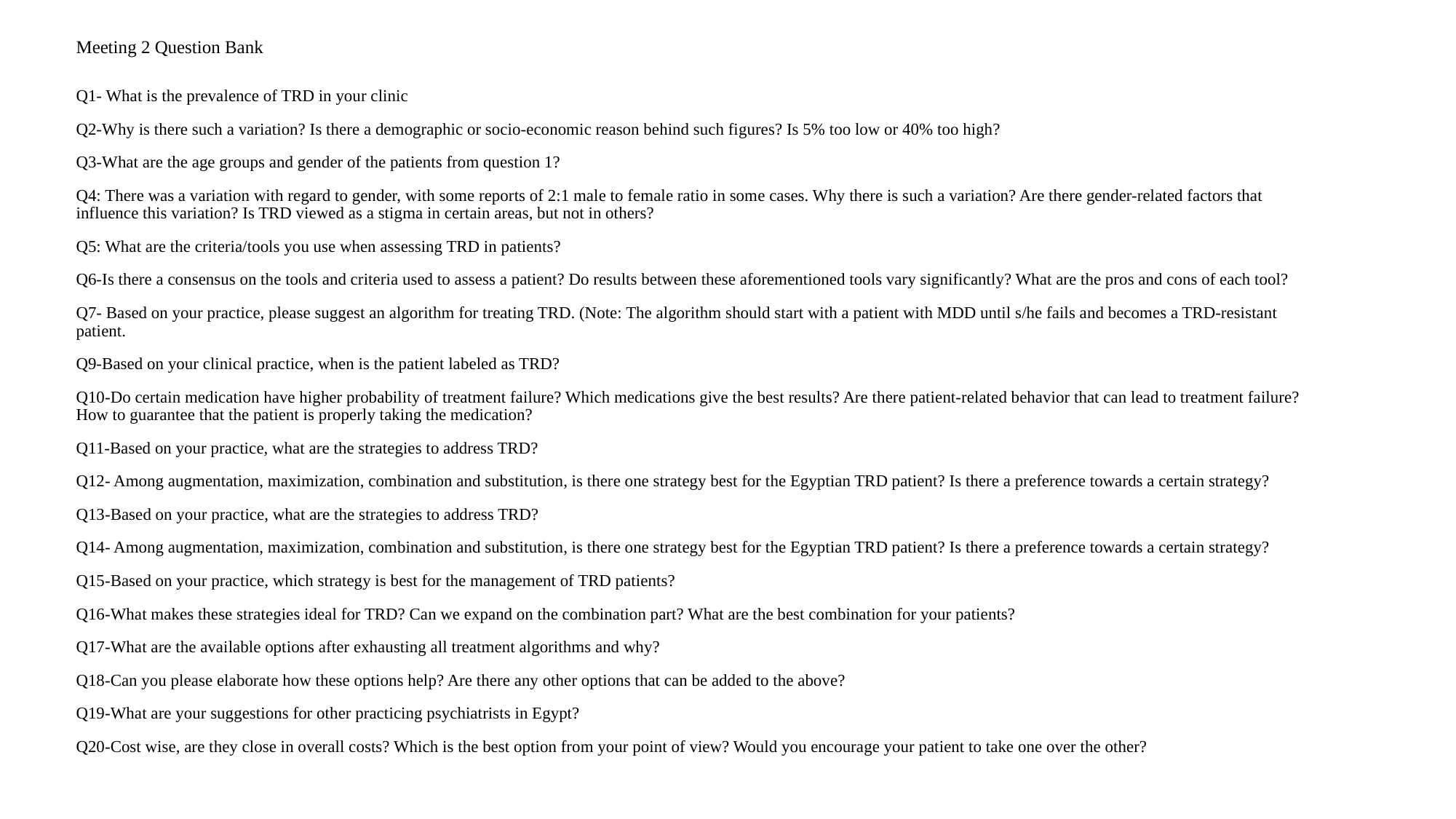

# Meeting 2 Question Bank
Q1- What is the prevalence of TRD in your clinic
Q2-Why is there such a variation? Is there a demographic or socio-economic reason behind such figures? Is 5% too low or 40% too high?
Q3-What are the age groups and gender of the patients from question 1?
Q4: There was a variation with regard to gender, with some reports of 2:1 male to female ratio in some cases. Why there is such a variation? Are there gender-related factors that influence this variation? Is TRD viewed as a stigma in certain areas, but not in others?
Q5: What are the criteria/tools you use when assessing TRD in patients?
Q6-Is there a consensus on the tools and criteria used to assess a patient? Do results between these aforementioned tools vary significantly? What are the pros and cons of each tool?
Q7- Based on your practice, please suggest an algorithm for treating TRD. (Note: The algorithm should start with a patient with MDD until s/he fails and becomes a TRD-resistant patient.
Q9-Based on your clinical practice, when is the patient labeled as TRD?
Q10-Do certain medication have higher probability of treatment failure? Which medications give the best results? Are there patient-related behavior that can lead to treatment failure? How to guarantee that the patient is properly taking the medication?
Q11-Based on your practice, what are the strategies to address TRD?
Q12- Among augmentation, maximization, combination and substitution, is there one strategy best for the Egyptian TRD patient? Is there a preference towards a certain strategy?
Q13-Based on your practice, what are the strategies to address TRD?
Q14- Among augmentation, maximization, combination and substitution, is there one strategy best for the Egyptian TRD patient? Is there a preference towards a certain strategy?
Q15-Based on your practice, which strategy is best for the management of TRD patients?
Q16-What makes these strategies ideal for TRD? Can we expand on the combination part? What are the best combination for your patients?
Q17-What are the available options after exhausting all treatment algorithms and why?
Q18-Can you please elaborate how these options help? Are there any other options that can be added to the above?
Q19-What are your suggestions for other practicing psychiatrists in Egypt?
Q20-Cost wise, are they close in overall costs? Which is the best option from your point of view? Would you encourage your patient to take one over the other?
